# The time has come for revising the rules of clozapine blood monitoring in Europe. A joint expert statement from the European Clozapine Task Force

**DOI:** 10.1192/j.eurpsy.2024.1816

**Published:** 2025-01-10

**Authors:** Hélène Verdoux, Robert A. Bittner, Alkomiet Hasan, Mishal Qubad, Elias Wagner, Alexis Lepetit, Manuel Arrojo-Romero, Christian Bachmann, Marieke Beex-Oosterhuis, Jan Bogers, Andreja Celofiga, Dan Cohen, Domenico de Berardis, Marc de Hert, Carlos de Las Cuevas, Bjørn H. Ebdrup, Konstantinos N. Fountoulakis, Daniel Guinart, Dolores Keating, Miloslav Kopeček, John Lally, Judit Lazáry, Jurjen J. Luykx, Olalla Maronas Amigo, Espen Molden, Jimmi Nielsen, Brian O’Donoghue, Pierre Oswald, Flavian S. Radulescu, Christopher Rohde, Marina Sagud, Emilio J. Sanz, Ivona Šimunović Filipčić, Iris E. Sommer, Heidi Taipale, Jari Tiihonen, Heli Tuppurainen, Selene Veerman, Alina Wilkowska, Edoardo Spina, Peter Schulte

**Affiliations:** 1 French Clozapine Task Force, University of Bordeaux, Inserm, Bordeaux Population Health Research Center, Bordeaux, France; 2 Goethe University Frankfurt, University Hospital, Department of Psychiatry, Psychosomatic Medicine and Psychotherapy, Frankfurt am Main, Germany; 3 Department of Psychiatry, Psychotherapy and Psychosomatics, Medical Faculty, University of Augsburg, Augsburg, Germany; 4 DZPG (German Center for Mental Health), Partner Site, Munich/Augsburg, Germany; 5 Evidence-based Psychiatry and Psychotherapy, Faculty of Medicine, University of Augsburg, Augsburg, Germany; 6 French Clozapine Task Force; ACPPA Group, Francheville, France; 7 Hospices Civils de Lyon, Hôpital des Charpennes, Villeurbanne, France; 8 Institut des Sciences Cognitives Marc Jeannerod, CNRS, Bron, France; 9 Department of Psychiatry, Complejo Hospitalario Universitario de Santiago, Santiago de Compostela, Spain; 10 Department of Child and Adolescent Psychiatry, Ulm University, Ulm, Germany; 11 Department of Hospital Pharmacy, Albert Schweitzer Hospital, Dordrecht, The Netherlands; 12 Mental Health Organization Rivierduinen, Leiden, The Netherlands; 13 Department of Psychiatry, University Medical Centre Maribor, Maribor, Slovenia; 14 Dutch Clozapine Collaboration Group, Alkmaar, The Netherlands; 15 Esdege-Reigersdaal, Heerhugowaard, The Netherlands; 16 NHS, Department of Mental Health, G. Mazzini Hospital, Teramo, Italy; 17 University Psychiatric Center Katholieke Universiteit Leuven, Kortenberg, Belgium; 18 Department of Neurosciences, Centre for Clinical Psychiatry, Katholieke Universiteit Leuven, Leuven, Belgium; 19 Leuven Brain Institute, Katholieke Universiteit Leuven, Leuven, Belgium; 20 Antwerp Health Law and Ethics Chair, University of Antwerp, Antwerp, Belgium; 21 Department of Internal Medicine, Dermatology and Psychiatry, School of Medicine, University of La Laguna, Canary Islands, Spain; 22 Center for Neuropsychiatric Schizophrenia Research (CNSR), Mental Health Center, Glostrup, Copenhagen University Hospital – Mental Health Services CPH, Copenhagen, Denmark; 23 Department of Clinical Medicine, Faculty of Health and Medical Sciences, University of Copenhagen, Copenhagen, Denmark; 24 3rd Department of Psychiatry, Division of Neurosciences, School of Medicine, Faculty of Health Sciences, Aristotle University of Thessaloniki, Thessaloniki, Greece; 25 Institut de Salut Mental, Hospital del Mar, Barcelona, Spain; 26 Programa Recerca en Salut Mental, Hospital del Mar Research Institute, CIBERSAM, Barcelona, Spain; 27 The Donald and Barbara Zucker School of Medicine, Northwell Health, New York, NY, USA; 28 Saint John of God Hospital, Stillorgan, Co. Dublin, Dublin, Ireland; 29 National Institute of Mental Health, Klecany, Czech Republic; 30 Department of Psychiatry, Third Faculty of Medicine, Charles University, Prague, Czech Republic; 31 Department of Psychiatry, School of Medicine and Medical Science, University College Dublin, Dublin, Ireland; 32 Department of Psychiatry, St Vincent’s Hospital Fairview, Dublin, Ireland; 33 Department of Psychosis Studies, Institute of Psychiatry, Psychology and Neuroscience (IoPPN), King’s College London, London, UK; 34 Nyírő Gyula National Institute of Psychiatry and Addictions, Budapest, Hungary; 35 Department of Psychiatry, Amsterdam UMC, Amsterdam, The Netherlands; 36 GGZ inGeest Mental Health Care, Amsterdam, The Netherlands; 37 Amsterdam Neuroscience (Mood, Anxiety, Psychosis, Stress & Sleep program) and Amsterdam Public Health (Mental Health program) Research Institutes, Amsterdam, The Netherlands; 38 Department of Psychiatry and Neuropsychology, School for Mental Health and Neuroscience Maastricht University Medical Center, Maastricht, The Netherlands; 39 Pharmacogenomics and Drug Discovery (GenDeM), Health Research Institute of Santiago de Compostela (IDIS), Santiago de Compostela, Spain; 40 Genomic Medicine Group, CIMUS, University of Santiago de Compostela, Santiago de Compostela, Spain; 41 Centre for Biomedical Network Research on Rare Diseases (CIBERER), Instituto de Salud Carlos III, Madrid, Spain; 42 Galician Public Foundation of Genomic Medicine (FPGMX), Galician Healthcare Service (SERGAS), Santiago de Compostela, Spain; 43 Section for Pharmacology and Pharmaceutical Biosciences, Department of Pharmacy, University of Oslo, Oslo, Norway; 44 Center for Psychopharmacology, Diakonhjemmet Hospital, Oslo, Norway; 45 Mental Health Centre Glostrup, Copenhagen University Hospital, Copenhagen, Denmark; 46 Department of Psychiatry, University College Dublin, Dublin, Ireland; 47 Department of Psychiatry, St Vincent’s University Hospital, Dublin, Ireland; 48 Department of Psychiatry, Hôpital Universitaire de Bruxelles, Bruxelles, Belgium; 49 Center for Drug Sciences, Faculty of Pharmacy, University of Medicine and Pharmacy Carol Davila, Bucharest, Romania; 50 Department of Affective Disorders, Aarhus University, Hospital - Psychiatry, Aarhus, Denmark; 51 Department of Clinical Medicine, Aarhus University, Aarhus, Denmark; 52 Department of Psychiatry, School of Medicine, University of Zagreb, Zagreb, Croatia; 53 Department for Psychiatry and Psychological Medicine, University Hospital Center Zagreb, Zagreb, Croatia; 54 Department of Physical Medicine and Pharmacology, School of Medicine, Universidad de La Laguna, Canary Islands, Spain; 55 Hospital Universitario de Canarias, Tenerife, Spain; 56 Department of Psychiatry and Psychological Medicine, University Hospital Centre Zagreb, Zagreb, Croatia; 57 Faculty of Dental Medicine and Health, Josip Juraj Strossmayer University of Osijek, Osijek, Croatia; 58 Department of Neuroscience, and Department of Psychiatry, University Medical Center Groningen, University of Groningen, Groningen, The Netherlands; 59 Department of Forensic Psychiatry, University of Eastern Finland, Niuvanniemi Hospital, Kuopio, Finland; 60 Department of Clinical Neuroscience, Karolinska Institutet, and Center for Psychiatry Research, Stockholm City Council, Stockholm, Sweden; 61 School of Pharmacy, University of Eastern Finland, Kuopio, Finland; 62 Neuroscience Center, University of Helsinki, Helsinki, Finland; 63 Mental Health Services Noord-Holland-Noord, Schagen, The Netherlands; 64 Dutch Clozapine Collaboration Group, Alkmaar, The Netherlands; 65 Department of Psychiatry, Medical University of Gdańsk, Gdańsk, Poland; 66 Department of Clinical and Experimental Medicine, University of Messina, Messina, Italy; 67 Mental Health Services Noord-Holland-Noord, Alkmaar, The Netherlands

**Keywords:** blood monitoring, clozapine, regulation, treatment-resistant schizophrenia

## Abstract

The European Clozapine Task Force is a group of psychiatrists and pharmacologists practicing in 18 countries under European Medicines Agency (EMA) regulation, who are deeply concerned about the underuse of clozapine in European countries. Although clozapine is the most effective antipsychotic for people with treatment-resistant schizophrenia, a large proportion of them do not have access to this treatment. Concerns about clozapine-induced agranulocytosis and stringent blood monitoring rules are major barriers to clozapine prescribing and use. There is a growing body of evidence that the incidence of clozapine-induced agranulocytosis is very low after the first year of treatment. Maintaining lifelong monthly blood monitoring after this period contributes to unjustified discontinuation of clozapine. We leverage recent and replicated evidence on the long-term safety of clozapine to call for the revision and updating of the EMA’s blood monitoring rules, thus aiming to overcome this major barrier to clozapine prescribing and use. We believe the time has come for relaxing the rules without increasing the risks for people using clozapine in Europe.

## Introduction

Shortly after the introduction of clozapine in the 70s, several cases of fatal agranulocytosis in Finland led to its market withdrawal in almost all countries. Following the study by Kane et al. in people with treatment-resistant schizophrenia (TRS) [1], clozapine was reintroduced with strict blood monitoring rules to detect incident agranulocytosis, estimated at 1–2% lifetime. More than 35 years later, these rules have not been revised in most countries [2]. Under current European Medicines Agency (EMA) regulation, white blood cell count (WBC) and absolute neutrophil count (ANC) monitoring is required weekly for the first 18 weeks after initiation of treatment and then monthly for the duration of treatment. For many patients, this can mean decades of monthly blood sampling.

Clozapine remains the only approved antipsychotic for TRS with superior efficacy and effectiveness for several endpoints, such as suicide, psychotic symptoms, relapse, rehospitalization, medication adherence, aggression, or substance use. Clozapine use is also associated with reduced all-cause, suicide, and cardiovascular mortality. While TRS occurs in about one-third of schizophrenia patients, only a minority of them are prescribed this medication [3], which represents a missed opportunity for these people, as their chances of recovery are much less favorable without clozapine [3–7].

The need for continued blood tests “for life” is a significant barrier to clozapine initiation and maintenance treatment increasing the risk of premature discontinuation [8, 9]. From the prescriber’s perspective, the most common barriers to clozapine initiation are related to the institutional complexity of mandatory blood monitoring and an overestimated users’ poor adherence to this monitoring [9]. Prescribers’ overestimation of agranulocytosis risk also contributes to under-monitoring of other adverse drug reactions with higher lethality including myocarditis, pneumonia, or ileus [10].

Blood monitoring also comes at a cost. In addition to the direct costs of blood tests, monitoring is time-consuming for laboratories and pharmacies. It requires complex organizational adaptations to guarantee continuity of care, particularly at a time when medical and paramedical staffing levels are low. A cost-effectiveness analysis compared several blood monitoring strategies in a theoretical cohort of 100,000 people treated with clozapine [10]. While the « no monitoring » strategy was the most cost-effective, no difference was found regarding quality-adjusted life-year gained compared to the other modalities, irrespective of the stringency of blood monitoring rules.

## Low risk of agranulocytosis after the first year of treatment

Recent studies have confirmed that the risk window for clozapine-related agranulocytosis is mostly limited to the first months of treatment. A meta-analysis of 108 studies (1983–2017, 448,647 clozapine users) found a 3.8% incidence of mild neutropenia (≤ 1.5 × 10^9^ per L), a 1.3% incidence of moderate neutropenia (≤ 1.0 × 10^9^ per L) and a 0.9% of severe neutropenia, commonly referred to as agranulocytosis (≤ 0.5 × 10^9^ per L) (0.7% when only high-quality studies were considered) [5]. The agranulocytosis fatality rate was one death for 7,700 clozapine users. The agranulocytosis prevalence was identical in studies conducted before and after the introduction of blood monitoring in 1990. In most cases (75%), mild neutropenia did not progress to severe neutropenia. Most cases of agranulocytosis occurred in the first few months of treatment: 38% in the first month, 56% within two months, 84% within the first 18 weeks, and 89% within the first year.

Neutropenia <1. 0 × 10^9^ per L occurred in 1.2% of users in a population-based study of 26,000 clozapine users in Australia and New Zealand (1990–2022) with no fatal cases [6]. The risk was highest during the first 18 weeks of treatment (weekly incidence 0.13%) and became negligible after 24 months. The incidence of any neutropenic event was very low when clozapine was reintroduced in people with no history of neutropenia over two years of cumulative monitoring. This finding suggests that there is no need to resume a weekly monitoring schedule after a clozapine interruption in people with no history of neutropenia.

Another population-based study of 14,037 clozapine users in Finland (1996–2017) found a cumulative incidence of agranulocytosis of 1.37% with clozapine compared with 0.13% with other antipsychotics [7]. The mortality rate for agranulocytosis was one death in 3,559 people starting clozapine. Compared to modal treatment duration for non-clozapine antipsychotics (12–23 months), the risk of agranulocytosis decreased over time from an adjusted odds ratio of 36 during the first six months of clozapine exposure to 4.38 for exposure ≥54 months: it became then comparable to that observed over the first six months of treatment for other antipsychotics for which no mandatory monitoring is required.

## Stringency of blood monitoring rules already differs between European countries

A review of clozapine monitoring regulations in 102 countries highlighted the wide variability in monitoring rules and in WBC/ANC criteria for stopping clozapine [2]. Although EMA regulation applies to all EU countries as well as Norway and Iceland, the blood monitoring rules already differ from one country to another [2] (Supplementary Table 1). In several countries, the rule “no blood, no drug” does not apply and monitoring is already relaxed. The recommendations of the Netherlands Clozapine Collaboration Group, set up in 2006, allow for off-label less stringent monitoring rules after the first 18 weeks of treatment if the prescriber and the well-informed patient decide so. Similar recommendations apply to Iceland with the possibility of relaxed monitoring after six months. Monitoring stringency has no impact on the incidence of agranulocytosis but is inversely associated with the rate of clozapine use in each country [2].

Criteria for clozapine discontinuation following a drop in WBC count also vary from country to country. Too stringent criteria may lead to unjustified discontinuation due to transient neutropenia not related to clozapine. Interdisciplinary boards involving psychiatrists and hematologists would be needed to exclude concurrent reasons for ANC decrease.

In most European countries, mild neutropenia, that is, ANC <1.5 × 10^9^ per L requires discontinuation (Supplementary Table 1). However, in a few European countries under EMA regulation, the US Food and Drug Administration (FDA) criteria (ANC <1.0 × 10^9^ per L) are used after 18 weeks [2].

Several countries have adjusted the threshold criteria for clozapine discontinuation for people concerned with benign ethnic neutropenia (BEN) [1] since their access to clozapine is markedly restricted under the standard criteria. For instance, the FDA threshold criteria for clozapine discontinuation for BEN is ANC <0.5 × 10^9^ per L. The BEN-adjusted criteria are not yet applied in any country under the EMA regulation, leading to discrimination regarding access to clozapine for the many people living with BEN in Europe. For example, up to 80% of people of black African or Caribbean descent have the Duffy-null phenotype of the ACKR1 (Atypical Chemokine Receptor 1) gene associated with BEN.

## Reducing barriers to clozapine by revising European blood monitoring rules

Thirty-five years ago, stringent blood monitoring rules were justified to ensure the successful reintroduction of clozapine. Today, this stringency no longer matches the reality of the actual hematological risk, as demonstrated by the growing body of evidence on the temporal pattern of clozapine-induced agranulocytosis. No other drug approved in Europe with a negligible risk of agranulocytosis after the first year of treatment requires lifelong monthly blood monitoring.

The European Clozapine Task Force is a group of psychiatrists and pharmacologists practicing in 18 countries under EMA regulation with extensive clinical and research expertise in clozapine. We are deeply concerned about the underuse of clozapine in European countries. We are convinced revising and updating the EMA’s blood monitoring rules could help to overcome the major barrier to the use of clozapine [9].

Our proposals for new clozapine blood monitoring rules are detailed in Table 1. Only ANC criteria are given as the majority of authors (60%) are in favor of restricting mandatory monitoring to ANC based on FDA regulation revisions in 2015. However, no consensus could be reached among the members of the European Clozapine Task Force on this point.

**Table 1.** European Clozapine Task Force proposals for new clozapine blood monitoring rules in countries under European Medicines Agency regulation

	Current SPC^1^	Proposals^2^
Mandatory routine blood monitoring schedule	WBC^3^ and ANC^4^ baseline before initiation weekly for 18 weeks after initiation then monthly irrespective of treatment duration	First 12 months ANC baseline before initiation weekly for 18 weeks after initiation then monthly for 34 weeks After 12 months^5^ ANC every 12 weeks if no history of leukopenia or neutropenia during the first year After 24 months^5^ yearly ANC if no history of leukopenia or neutropenia during two years
Standard thresholds for		
Initiation/continuation	ANC ≥ 2.0 x 10^9^/L WBC ≥ 3.5 x 10^9^/L	ANC ≥ 1.5 × 10^9^ per L^6^
Monitoring twice a week	ANC 1.5–2 x 10^9^/L WBC 3.0–3.5 x 10^9^/L	ANC 1.0–1.5 x 10^9^/L^6^
Discontinuation (red)	ANC <1.5x10^9^/L WBC <3.0x10^9^/L	ANC < 1.0 × 10^9^ per L^6^
BEN^7^ adjusted thresholds for		
Initiation/continuation		ANC ≥ 1.0 × 10^9^ per L^6^
Monitoring twice a week		ANC 0.5–1.0× 10^9^ per L^6^
Discontinuation		ANC < 0.5 × 10^9^ per L^6^
Monitoring schedule after clozapine interruption	interruption > 3 days and < 4 weeks weekly for 6 weeks then monthly interruption > 4 weeks weekly for 18 weeks then monthly	irrespective of the duration of interruption no need to resume weekly schedule if no history of neutropenia during two cumulative years of monitoring

1 Summaries of Product Characteristics;

2 Only Absolute Neutrophil Count criteria are given as the majority of authors (60%) are in favor of restricting mandatory monitoring to ANC based on Food and Drug Administration regulation revisions in 2015; however, no consensus could be reached among the members of the European Clozapine Task Force on this point;

3 White Blood Cells count;

4 Absolute Neutrophil Count;

5 Even if the frequency of routine mandatory monitoring is reduced, ANC must be performed immediately in the event of possible symptoms of infection (e.g. fever, sore throat, mouth/throat ulcers). Additional ANC may be considered after addition of valproic acid to clozapine, especially during the initiation period.

6 Food and Drug Administration criteria;

7 Benign Ethnic Neutropenia (hematology consultation may be needed to confirm the diagnosis).

Based on the currently available evidence, we propose to:

- relax the blood monitoring schedule after 12 months and 24 months of treatment,
- lower ANC threshold for clozapine initiation and discontinuation,
- use an adjusted threshold for BEN,
- relax monitoring schedule after clozapine interruption,
- harmonize (i) to (iv) across Europe.

The benefits of revising the monitoring rules outweigh the potential risks associated with less stringent rules: allowing more people with TRS to get clozapine will save lives without increasing the risk of agranulocytosis.

## Conclusion

Access to clozapine is currently severely hampered by multiple barriers resulting in a real loss of opportunity for many people with TRS, with a high price being paid by both the users and the healthcare system. Whenever deemed indicated, clozapine treatment must be initiated as soon as possible to promote recovery and to increase life expectancy. Better addressing these unmet needs should be considered as a public health priority by European health regulatory agencies, as it is currently done by the FDA. More balanced monitoring rules would contribute to reducing clozapine underprescription as well as discrimination against people with BEN. The time has come for revising the rules of clozapine blood monitoring in Europe.

## Supporting information

Verdoux et al. supplementary material Verdoux et al. supplementary material

## Data Availability

Not relevant.

## Supplementary material

The supplementary material for this article can be found at http://doi.org/10.1192/j.eurpsy.2024.1816.
