# Supplementary material for: The time has come for revising the rules of clozapine blood monitoring in Europe. A joint expert statement from the European Clozapine Task Force
Source: Eur Psychiatry. 2025 Jan 10;68(1):e17. doi: 10.1192/j.eurpsy.2024.1816 (PMC11822956; doi:10.1192/j.eurpsy.2024.1816)
Supplement: Verdoux et al. supplementary material [file S0924933824018169sup001.docx]

**Supplementary Table 1. Clozapine hematological monitoring rules in countries with European Medicines Agency regulation**

**(adapted from Oloyede et al. 2022) [2]**

| **Country** | **“no blood no drug”** | **Frequency (weeks)** | | | **Criteria for clozapine discontinuation** | |
| --- | --- | --- | --- | --- | --- | --- |
|  |  | **weekly** | **Fortnightly** | **Monthly** | **WBC^1^ (****mm^3^/L)** | **ANC^2^ (mm^3^/L)** |
| **Austria** | YES | 18 wk |  | >19 wk | <3.0 | <1.5 |
| **Belgium** | YES | 18 wk |  | >19 wk | <3.0 | <1.5 |
| **Bulgaria** | NO | 4 wk |  | > 4 wk | NO | NO |
| **Croatia** | YES | 18 wk |  | >19 wk | <3.0 | <1.5 |
| **Cyprus Republic** | YES | 18 wk |  | >19 wk | NO | NO |
| **Czech Republic** | YES | 18 wk |  | >19 wk | <3.0 then <2.5^3^ | <1.5 then <1.0^3^ |
| **Denmark** | YES | 18 wk |  | >19 wk | <3.0 | <1.5 |
| **Estonia** | YES | 18 wk |  | >19 wk | <3.0 | <1.5 |
| **Finland** | NO | 18 wk |  | >19 wk | <3.0 | <1.5 |
| **France** | YES | 18 wk |  | >19 wk | <3.0 | <1.5 |
| **Germany** | YES | 18 wk |  | >19 wk | <3.0 | <1.5 |
| **Greece** | YES | 18 wk |  | >19 wk | <3.0 | <1.5 |
| **Hungary** | YES | 18 wk |  | >19 wk | <3.0 | <1.5 |
| **Iceland^4^** | NO | 18 wk |  | >19 wk | <3,0 | <1,5 |
| **Ireland** | YES | 18 wk | 19-51 wk | > 52 wk | <3.0 | <1.5 |
| **Italy** | YES | 18 wk |  | >19 wk | <3.0 | <1.5 |
| **Latvia** | YES | 18 wk |  | >19 wk | <3.0 | <1.5 |
| **Lithuania** | YES | 18 wk |  | >19 wk | <3.0 | <1.5 |
| **Luxembourg** | YES | 18 wk |  | >19 wk | <3.0 | <1.5 |
| **Malta** | YES | 18 wk | 19-51 wk | > 52 wk | <3.0 | <1.5 |
| **Netherlands^5^** | YES | 18 wk |  | >19 wk | <3.0 | <1.5 |
| **Norway** | YES | 18 wk |  | >19 wk | <3.0 | <1.5 |
| **Poland** | NO | 18 wk |  | >19 wk | <3.0 | <1.5 |
| **Portugal** | NO | 18 wk |  | >19 wk | <3.0 | <1.5 |
| **Romania** | YES | 18 wk |  | >19 wk | <3.0 | <1.5 |
| **Slovakia** | YES | 18 wk |  | >19 wk | <3.0 then <2.5^3^ | <1.5 then <1.0^3^ |
| **Slovenia** | YES | 18 wk |  | >19 wk | <3.0 | <1.5 |
| **Spain** | NO | 18 wk |  | >19 wk | <3.0 | <1.5 |
| **Sweden** | YES | 18 wk |  | >19 wk | <3.0 | <1.5 |

1. White Blood Cells count ; 2. Absolute Neutrophil Count ; 3. after 18 weeks ; 4. relaxed rules are most often applied ; 5. the Dutch clozapine guideline permits relaxed rules after the first months of treatment if the prescriber and the well-informed patient decide so.
